# OLD family nuclease function across diverse anti-phage defense systems

**DOI:** 10.3389/fmicb.2023.1268820

**Published:** 2023-09-28

**Authors:** Konstantina Akritidou, Bryan H. Thurtle-Schmidt

**Affiliations:** Department of Biology, Davidson College, Davidson, NC, United States

**Keywords:** OLD nuclease, overcoming lysogenization defect, Gabija, retrons, Toprim, ABC ATPase, anti-phage defense, abortive infection

## Abstract

Bacteriophages constitute a ubiquitous threat to bacteria, and bacteria have evolved numerous anti-phage defense systems to protect themselves. These systems include well-studied phenomena such as restriction endonucleases and CRISPR, while emerging studies have identified many new anti-phage defense systems whose mechanisms are unknown or poorly understood. Some of these systems involve overcoming lysogenization defect (OLD) nucleases, a family of proteins comprising an ABC ATPase domain linked to a Toprim nuclease domain. Despite being discovered over 50 years ago, OLD nuclease function remained mysterious until recent biochemical, structural, and bioinformatic studies revealed that OLD nucleases protect bacteria by functioning in diverse anti-phage defense systems including the Gabija system and retrons. In this review we will highlight recent discoveries in OLD protein function and their involvement in multiple discrete anti-phage defense systems.

## 1. Introduction

Bacteria are under constant threat from viruses termed bacteriophages, or phages. It is estimated that there are 10^31^ phage particles in nature (Hendrix et al., 1999; Mushegian, 2020), making them the most abundant biological agent on the planet. To protect themselves from this ubiquitous threat, bacteria have evolved numerous systems to ward off phage infections (Hampton et al., 2020; Georjon and Bernheim, 2023). A recurring theme in many of these defense systems is the targeted cleavage of phage nucleic acid. The restriction-modification (R-M) system constitutes a classic example (Loenen et al., 2014), while more recently CRISPR sequences were discovered to generate immunological memory of previous infections and ultimately generate acquired defense (Mojica et al., 2005; Barrangou et al., 2007). Similar to how the R-M system once ushered in the modern era of recombinant DNA technology, the CRISPR/Cas9 system has likewise revolutionized biological and industrial research.

The impact of R-M and CRISPR/Cas9 systems demonstrates the fruitfulness of basic biological research into anti-phage defense mechanisms, and recent years have witnessed the discovery of myriad anti-phage defense systems, many of which remain relatively uncharacterized. Some anti-phage defense systems feature the activity of prokaryotic Argonaute proteins that employ DNA endonuclease activity as the driving mechanism of anti-phage defense (Swarts et al., 2014; Kuzmenko et al., 2020). Others, like BREX (bacteriophage exclusion; Goldfarb et al., 2015), involve methylation to distinguish self from non-self DNA but do not rely on nucleolytic degradation to achieve cell defense (Gordeeva et al., 2019). Other systems do not achieve defense through the preservation of the cell but rather through abortive infection, in which infected cells effect cell death before the phage can complete its replicative cycle (Lopatina et al., 2020). Systems resulting in abortive infection include CBASS (cyclic oligonucleotide-based antiphage signaling system) and PYCSAR (pyrimidine cyclase system for antiphage resistance) systems, which use cyclic dinucleotides as signaling molecules (Cohen et al., 2019; Tal et al., 2021). Systematic surveys of genomes, in particular focusing on genomic defense islands, continue to uncover new defense systems (Doron et al., 2018; Gao et al., 2020; Millman et al., 2022), most of which remain poorly understood. A close look at several systems, including the Gabija system (Doron et al., 2018; Cheng et al., 2021) and retrons (Millman et al., 2020), illuminate a recurring appearance of overcoming lysogenization defect (OLD) family nucleases. A classification scheme has been proposed in which OLD proteins can be assigned to different classes depending on their surrounding genetic context (Dot et al., 2023). In this review we will focus on the composition, structure, and function of Class 1, Class 2, and Class 3 OLD nucleases across diverse anti-phage defense systems.

## 2. Class 1 OLD proteins: phage-phage interference and structure overview

**Figure 1:**
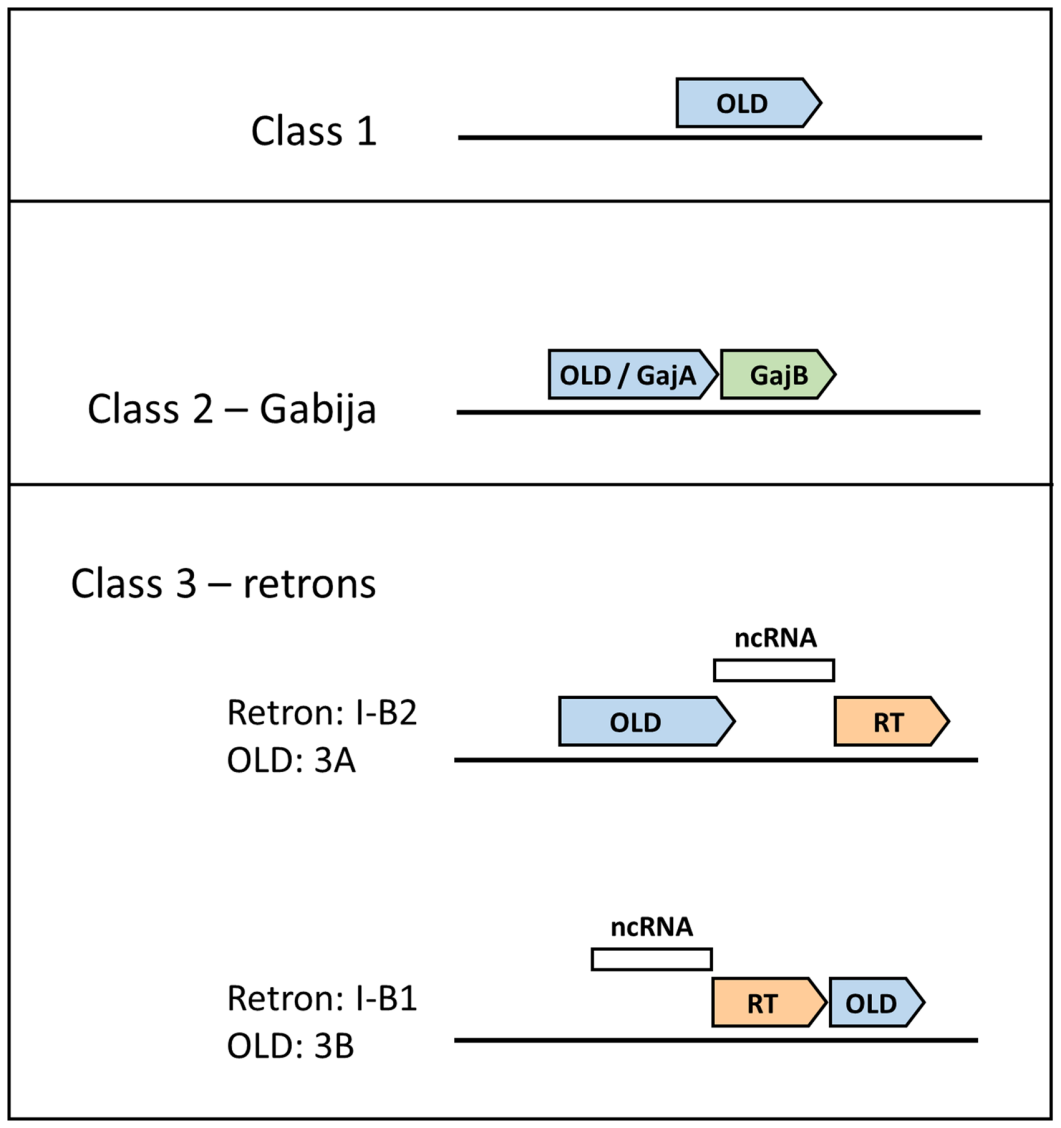
Genomic layouts of OLD protein classes. Gene neighborhood organizations for Class 1, 2, and 3 OLD proteins are shown, with OLD proteins in blue. Class 1 OLD proteins appear as single genes. Class 2 OLD proteins are synonymous with GajA, and are found together with GajB (green), which shows homology to UvrD/PcrA/Rep-like helicases. Class 3 OLD proteins are found in retron cassettes and have two possible genomic layouts depending on OLD positioning relative to the reverse transcriptase (RT, orange) and non-coding RNA (ncRNA, white). Class 3A OLD proteins are found in type I-B2 retrons, while Class 3B OLD proteins are found in type I-B1 retrons.

Although defense islands have been increasingly observed to harbor anti-phage defense systems (Makarova et al., 2011), Class 1 OLD systems belie this trend as they are instead composed of single genes not found proximal to other candidate defense genes (Figure 1; Schiltz et al., 2019). The archetype for understanding Class 1 OLD proteins, and indeed the original discovery and namesake for the entire OLD family of proteins, arises from early phage genetics experiments. Gianpiero Sironi showed that P2 phage was unable to lysogenize *E. coli* mutants he named *lyd* (lysogenization defective), and subsequently identified P2 mutants that could lysogenize *lyd* mutants (Sironi, 1969). Sironi named this P2 mutant phenotype *old* for overcoming lysogenization defect. Subsequent work determined that lambda phage is unable to lysogenize a wild-type P2 prophage but that this phage interference is eliminated by mutations in *old* (Lindahl et al., 1970). *Lyd* mutants turned out to reside in *recB* and *recC* (Lindahl et al., 1970) components of the RecBCD helicase-nuclease complex that plays critical roles both in homologous recombination and in defense against phages *via* double-stranded DNA degradation (Dillingham and Kowalczykowski, 2008). Expression of the P2 Old protein is sufficient to kill *recBC*^−^ cells (Sironi et al., 1971), a phenotype that continues to be used to assess P2 Old function (Schiltz et al., 2020). P2 Old’s interference with lambda phage was an early example of prophage-encoded anti-phage defense, a phenomenon that is now recognized as widespread (Patel and Maxwell, 2023). Indeed, the P2 *old* locus has been observed to encode other anti-phage systems at this position (Rousset et al., 2022; Vassallo et al., 2022).

**Figure 2:**
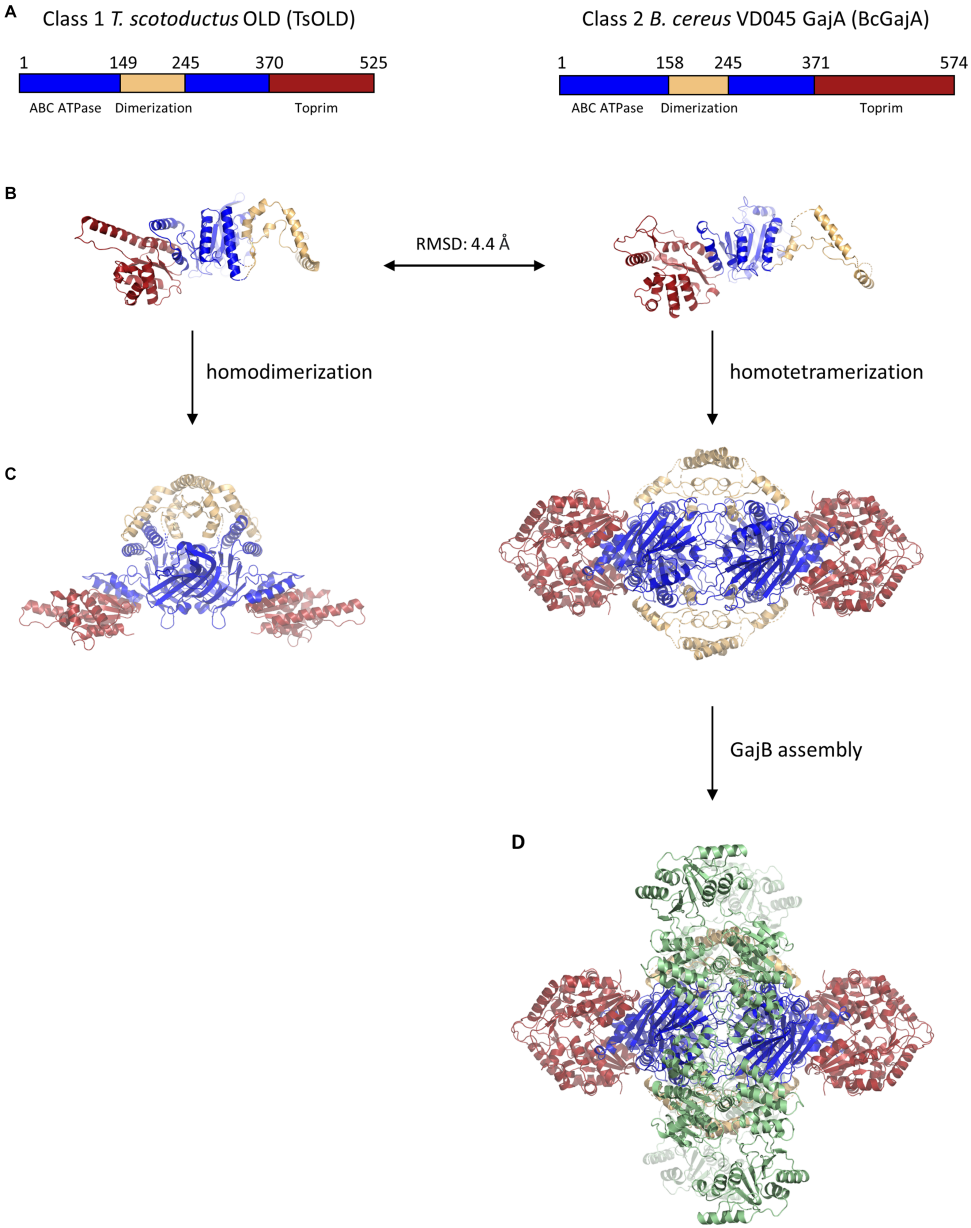
Structural similarity and multimeric assembly of Class 1 and Class 2 OLD proteins. **(A)** Schematics showing domain organization within Class 1 *T. scotoductus* OLD and Class 2 *B. cereus* GajA OLD proteins. A dimerization domain (tan) is inserted into each ABC ATPase domain (blue). The larger size of BcGajA arises from a helical insert into the Toprim domain (red) that is conserved among Class 2 OLD proteins and makes them larger than Class 1 OLD proteins by about 50 amino acids on average. **(B)** Structural superposition of TsOLD (PDB: 6P74) and BcGajA (PDB: 8SM3) full-length monomers shows structural similarity (RMSD = 4.4 Å) despite only 22.2% sequence identity. **(C)** TsOLD dimerizes and BcGajA tetramerizes in crystal structures, but in neither structure are the ATPases poised for ATP hydrolysis. **(D)** Two separate GajB dimers (green) bind to a GajA homotetramer to assemble an octameric GajAB complex.

After early phage genetics defined the protein name and function in P2, studies of OLD nucleases mostly disappeared for many years. One notable exception was the purification and characterization of a P2 Old construct fused to maltose-binding protein (MBP; Myung and Calendar, 1995). This study established that P2 Old-MBP displayed 5’to 3′ exonuclease activity on dsDNA. Furthermore, ATP was found to enhance but not be required for DNA cleavage, and the ATPase activity was not stimulated by the addition of DNA. Whether these activities were unique to P2 Old or were common to OLD proteins would remain unknown until recently (Table 1). A few years later, bioinformatic analysis showed that OLD proteins were composed of an N-terminal ATP-binding cassette (ABC)-family ATPase and a C-terminal Toprim domain (Figure 2; Aravind et al., 1998). ABC ATPases are found in diverse proteins ranging from membrane transporters to nuclear structural maintenance of chromosomes (SMC) proteins including condensins and cohesins (Krishnan et al., 2020). The Toprim domain is a divalent metal-binding domain found in topoisomerases, DnaG-type primases, RecR proteins, and OLD family nucleases (Aravind et al., 1998). Toprim domains possess a conserved acidic motif that binds to divalent cations which promote phosphoryl transfer reactions (Keck et al., 2000; Kato et al., 2003; Schmidt et al., 2010), suggesting that DNA cleavage by OLD proteins may follow canonical two-metal DNA cleavage mechanisms (Steitz and Steitz, 1993).

**Table 1:** Functional properties of OLD protein classes.

OLD class	Surrounding genetic context	Select examples	Targeted phages	Structural data	DNA cleavage activities	Nucleotide effect on DNA cleavage	ATP hydrolysis activity?
Class 1	Single genes	P2 Old (UniProt: P13520)	Lambda	None	Nonspecific 5′ to 3′ exonuclease	Higher [ATP] causes slight increase in DNA cleavage	Yes
		*T. scotoductus* OLD (UniProt: E8PLM2)	Unknown	Homodimeric assembly (PDB: 6P74)	Nonspecific 5′ to 3′ exonuclease; linearizes circular DNA	Negligible	Yes
Class 2 Gabija	Adjacent to UvrD/PcrA/Rep-like helicase	*B. cereus* VD045 GajA (UniProt: J8H9C1)	SBSphiC, SPβ, Φ105, rho14, Φ29	Octamer with 4:4 ratio of GajA:GajB (PDB: 8SM3)	Site-specific nicking Activity	Higher [ATP] strongly inhibits DNA cleavage	No
		*B. pseudomallei* OLD (UniProt: A3NFC3)	Unknown	Isolated Toprim domain (PDB: 6NK8)	nonspecific 5′ to 3′ exonuclease; linearizes circular DNA	Negligible	Yes
Class 3 retrons	Upstream (3A) or downstream (3B) from reverse transcriptase and non-coding RNA	*E. coli* 200,499 Retron-Eco8 (UniProt: P0DV58)	T4, T7, SECΦ4, SECΦ6, SECΦ18	None	Unknown	Unknown	Unknown

Decades would pass until the field of OLD nuclease research was reignited recently with detailed studies of OLD nuclease structure and function, as well as bioinformatic and genetic analyses of their function in anti-phage defense systems. A critical breakthrough was the first structural determination of a full-length OLD protein, the Class 1 OLD in *Thermus scotoductus* (TsOLD; Figure 2; Schiltz et al., 2020). The structure revealed the protein to adopt a homodimeric structure in which dimerization was mediated through a dimerization domain inserted into the ABC ATPases. Although the ATPases are docked in a conformation not competent to achieve ATP hydrolysis, the structure confirmed that the ATPase domain has structural homology with the ATP-binding cassette (ABC) family of ATPases. Further structural studies will be required to determine whether Class 1 OLD proteins may exist in higher multimeric complexes or associate with other proteins. Biochemical characterization of TsOLD showed that, like P2 Old, TsOLD displays robust 5’to 3′ exonuclease activity on linear dsDNA, as well as the ability to linearize circular plasmid DNA substrates. However, TsOLD is unlike P2 OLD in that the addition of ATP has essentially no effect on DNA cleavage by TsOLD (Table 1; Schiltz et al., 2020).

Mutagenesis studies in P2 Old showed that the conserved acidic metal-binding residues of the Toprim domain, as well as the conserved Walker A lysine critical for ATP binding, were all required for killing *recBC*^−^ cells, implicating both the ATPase and Toprim domains as essential for at least some *in vivo* activities (Schiltz et al., 2020). However, although the structural determination of TsOLD was a watershed moment propelling OLD protein research forward, the only Class 1 OLD protein established to provide defense against a phage remains P2 Old’s defense of prophages against lambda. The anti-phage defense functions of OLD proteins are better understood from studies of multicomponent systems featuring Class 2 or Class 3 OLD proteins.

## 3. Class 2 OLD proteins: the Gabija system

Recent discoveries of diverse anti-phage defense systems show that OLD proteins not only function on their own as Class 1 proteins but also are found in multicomponent anti-phage defense systems such as Class 2 OLDs found in the Gabija system (Doron et al., 2018). The genetic organization of the Gabija system was discovered independently by two groups. In the first instance, as part of a systematic discovery of anti-phage defense systems, the Sorek group identified a system composed of an OLD protein (GajA), and a UvrD/PcrA/Rep-like helicase (GajB), a system they would name Gabija after the Lithuanian mythology goddess of fire (Figure 1; Doron et al., 2018). They estimated the Gabija system is present in 8.5% of a set of 38,167 microbial genomes, while more recent bioinformatic tools designed to identify anti-phage defense systems place Gabija frequency closer to 15% (Payne et al., 2022; Tesson et al., 2022). The second Gabija system discovery came from the Chappie group which was studying OLD nucleases and termed them as Class 1 or Class 2 depending on whether they were found alone (Class 1) or in tandem with a UvrD/PcrA/Rep-like helicase (Class 2; Schiltz et al., 2019).

The first structural work on Class 2 OLD nucleases was performed on isolated Toprim domains from *Burkholderia pseudomallei* (BpOLD) and *Xanthomonas campestris* p.v. *campestris* (XccOLD; Schiltz et al., 2019). Their Toprim domains contain an extra helical domain insert that differentiates them from Class 1 OLD proteins and makes Class 2 OLD proteins about 50 amino acids longer on average (Figure 2). Structural analysis of the BpOLD Toprim domain shows its conserved acidic motif binds two Mg^2+^ ions and suggests a canonical two-metal mechanism for DNA cleavage. Biochemical assays with full-length BpOLD and XccOLD show nonspecific endonuclease and exonuclease activity on lambda phage DNA which is unaffected by ATP concentration (Table 1). The authors proposed that the BpOLD ATPase domain, which is competent to hydrolyze ATP (Cheng et al., 2021), plays a regulatory role in the cleavage of substrates by the Toprim domain (Schiltz et al., 2019).

The most studied Gabija system is from *Bacillus cereus* VD045 and is found to offer protection against phages of the *Siphoviridae* family including the SPβ, Φ105, and rho14, as well as phages from the *Podoviridae* family such as Φ29 (Table 1; Doron et al., 2018). Although initial biochemical characterization described the *B. cereus* GajA (BcGajA) as functionally distinct from OLD nucleases (Cheng et al., 2021), structural superposition of full-length monomers from *T. scotoductus* OLD and BcGajA reveals an RMSD of 4.4 Å despite a sequence identity of only 22.2% (Figure 2; Schiltz et al., 2020; Antine et al., 2023). On the basis of their structural similarity and their shared genomic proximity to UvrD/PcrA/Rep-like helicases, we suggest that all GajA proteins are Class 2 OLD proteins, and vice versa.

Like all Class 2 OLD proteins, BcGajA comprises an N-terminal ABC ATPase domain and a C-terminal Toprim domain. BcGajA is currently the only OLD protein known to cleave DNA with sequence specificity (Table 1), as it nicks DNA at a site found both in lambda and T7 phage dsDNA (Cheng et al., 2021). Interestingly, in lambda DNA the two cut sites overlap, resulting in apparent dsDNA cleavage activity. It is worth noting that BcGajA resides in a gram-positive bacteria that is not infected by lambda or T7 phages, and thus the significance of this cut site being found in lambda and T7 phage DNA is not clear. BcGajA endonuclease activity is robust under low ATP concentrations, while high nucleotide concentrations inhibit BcGajA DNA binding and cleavage (Cheng et al., 2021, 2023). Interestingly, BcGajA is the first OLD protein shown to lack ATP hydrolysis activity (Table 1). These results led the authors to propose that the ATPase domains regulate the Toprim domain by inhibiting its DNA cleavage activity in the presence of high nucleotide concentrations and that GajA becomes activated upon nucleotide depletion resulting from phage invasion, replication, and transcription. Recent studies have reported nucleotide depletion mechanisms in other anti-phage defense systems (Hsueh et al., 2022; Tal et al., 2022), supporting the idea that nucleotide depletion may be common in abortive infection mechanisms.

The second component of the Gabija system, GajB, is predicted to be a UvrD/PcrA/Rep-like helicase (Doron et al., 2018). UvrD helicases translocate along ssDNA in the 3′ to 5′ direction and couple the binding and hydrolysis of one ATP with the unwinding of one base-pair of duplex DNA (Matson, 1986; Lee and Yang, 2006). Recent structural and biochemical data have shed light on the function of GajB and its interactions with GajA. Structural studies show that BcGajB binds to a pre-formed BcGajA tetramer to assemble a 4:4 octameric complex in which with two sets of GajB dimers flank a centralized GajA tetramer (Figure 2; Antine et al., 2023). Although BcGajA purified in the absence of BcGajB displays robust endonuclease nicking activity (Cheng et al., 2021), BcGajB is required for anti-phage defense via an abortive infection mechanism (Doron et al., 2018; Cheng et al., 2023). A recent study shows that BcGajB, surprisingly, does not exhibit any helicase activity but instead functions as a (d)ATP/(d)GTPase (Cheng et al., 2023). Furthermore, the addition of either ssDNA or dsDNA stimulates nucleotide hydrolysis by GajB. The authors propose that BcGajB senses 3′ termini, possibly originating from BcGajA DNA cleavage, which activates its (d)ATP/(d)GTPase hydrolytic activity, thereby driving nucleotide depletion and contributing to cell death (Cheng et al., 2023). Further studies will be required to see whether these results, including a lack of GajB helicase activity, are generalizable to other Gabija systems.

## 4. Class 3 OLD proteins: retron-driven anti-phage defense

Retrons are genetic elements found in bacteria comprising a reverse transcriptase and an adjacent non-coding RNA. Their discovery stems from a 1984 study identifying a small multi-copy, single-stranded DNA (msDNA; Yee et al., 1984), which subsequent studies determined was composed of a ssDNA covalently linked to a non-coding RNA used as a template by the reverse transcriptase (Dhundale et al., 1987; Lampson et al., 1989). Retron function remained mysterious until a recent breakthrough study that demonstrated retrons belong to anti-phage defense systems triggering cell death through abortive infection (Millman et al., 2020). These defense systems contain three components: the aforementioned reverse transcriptase and non-coding RNA, as well as an effector protein (Figure 1). Effector proteins vary tremendously and include cold shock proteins, zinc finger nucleases, and proteases. Out of 4,802 genomes analyzed, the retron effector protein was an OLD nuclease 4% of the time (Millman et al., 2020). OLD nucleases found as effector proteins in retron defense systems have been classified as Class 3 OLD enzymes, with further subdivision depending on whether the *old* gene lies upstream (Class 3A) or downstream (Class 3B) of the reverse transcriptase (Dot et al., 2023). Within the retron naming system, these genomic organizations have been described as type I-B2 or type I-B1, respectively (Mestre et al., 2020).

Class 3 OLD enzymes have not yet been characterized through biochemical and structural means like either Class 1 or Class 2 OLD enzymes, but AlphaFold models show that, like their Class I and Class 2 counterparts, they comprise an N-terminal ABC ATPase and a C-terminal Toprim domain. Most of what is known about Class 3 OLD proteins arises from genetic studies of anti-phage defense. The best characterized Class 3 OLD protein belongs to Retron-Eco8, which provides defense against T4, T7, SECΦ4, SECΦ6, and SECΦ18 through an abortive infection mechanism (Table 1; Millman et al., 2020). Mutational analysis shows that mutations in the conserved aspartates in the YADD motif of the reverse transcriptase, or in the conserved guanosine branching point in the non-coding RNA, or in the Walker A lysine of the OLD nuclease each eliminate anti-phage defense activity (Millman et al., 2020). These data demonstrate that all three retron components are necessary for anti-phage defense by Retron-Eco8.

The mechanisms by which retrons in general and Class 3 OLD enzymes in particular lead to either phage recognition or abortive infection remain elusive. A recent study shows that T7, SECΦ4, SECΦ6, and SECΦ18 phages were all able to escape anti-phage defense by Retron-Eco8 through mutations in phage single-stranded binding (SSB) protein (Stokar-Avihail et al., 2023). This study showed that expressing the phage SSB in cells that express Retron-Eco8 was sufficient to drive cell toxicity and that mutations in the SSB were sufficient to alleviate that toxicity. Furthermore, pull-down experiments showed that wild-type but not mutant phage SSB was pulled down with Retron-Eco8 msDNA. While these data demonstrate a direct association between retron msDNA and phage SSB, they do not explain the role of the Class 3 OLD enzyme, which was already established to be essential for anti-phage defense (Millman et al., 2020). Further studies of Class 3 OLD enzymes will be required to understand their role in mediating retron-dependent anti-phage defense.

## 5. Future considerations

The last several years have seen a marked increase in studies examining the structure, biochemistry, and *in vivo* function of OLD proteins. The results of these studies reveal a complicated landscape in which the biochemical properties and functions of OLD proteins can vary from one another and depend heavily on the surrounding genetic, cellular, and chemical context. Many outstanding questions in the field remain, and below we highlight just a few of them:

*Do Class 1 OLD proteins provide broad-spectrum anti-phage defense on their own?* The only known example of a Class 1 OLD protein offering protection against a phage is the idiosyncratic example of P2 prophages providing Old-dependent defense only against lambda phage. Although the broad-spectrum anti-phage defense by Class 2 (Gabija) and Class 3 (retron) OLD proteins has been definitively established, there remains no known example of a bacterial or archaeal Class 1 OLD protein sufficient to drive broad-spectrum anti-phage defense. Multicomponent systems like Gabija and retrons have shown that each genetic component is necessary for anti-phage defense, and so it would be valuable to learn of cellular systems where an OLD protein alone is sufficient to drive anti-phage defense.

*What are the regulatory relationships between OLD ATPases, Toprim domains, and DNA engagement?* It may be that the answer depends on the specific OLD protein in question. For example, ATP has been reported to increase DNA cleavage for P2 Old, have minimal effect on DNA cleavage in TsOLD, and strongly inhibit DNA cleavage activity in BcGajA (Table 1). More work will be required to understand the regulatory relationships between the ATPase and Toprim domains. Moreover, although structural studies of OLD proteins have been critical breakthroughs, there remains a paucity of structural data of OLD proteins bound to DNA. Such studies will be required to understand how the protein engages with DNA, the physical basis for any sequence- or structural specificity OLD proteins may have, and what regulatory mechanisms might prevent DNA binding or cleavage depending on the surrounding biochemical context.

*How do phages escape these defense systems?* While bacteria have evolved complex mechanisms to impede viral infection, phages continually develop mechanisms to overcome them (Gao and Feng, 2023). One such mechanism involves the release of the Gabija anti-defense 1 (Gad1) protein by phage Φ3T to thwart the Gabija system of *B. cereus* VD045 (Yirmiya et al., 2023). To resist anti-phage defense, Gad1 binds to the GajA dimerization domain of the GajAB complex and prevents DNA binding and cleavage (Antine et al., 2023). Similar phage proteins called Thoeris anti-defense 1 and 2 (Tad1 and Tad2) have been recently discovered in the Thoeris system, another widely distributed bacterial anti-phage defense system (Leavitt et al., 2022). Future studies will no doubt illuminate other escape mechanisms, the understanding of which may be critical for the development of successful phage therapy (Kortright et al., 2019).

*How many classes of OLD proteins exist, and in how many different systems?* Class 4 OLD proteins have been proposed to contribute to the function of the PARIS system (Rousset et al., 2022; Dot et al., 2023). Unlike the other three classes, in the PARIS system the ATPase and Toprim domains are encoded on separate genes (designated ariA and ariB, respectively; Rousset et al., 2022). However, the authors noted that sometimes AriAB is encoded as a single-gene fusion, in which case it is not clear whether the system comprises a separate OLD class, or whether the system operates differently from Class 1 OLD proteins. Further studies will be required to determine whether the PARIS system constitutes a separate OLD class. Another recent study noted that a component of the anti-plasmid Wadjet system (Doron et al., 2018), jetD, has homology with the Toprim domain of OLD nucleases, while the jetABC components include an ABC ATPase with homology to the bacterial condensin complex MukBEF (Deep et al., 2022). These studies suggest we have much to learn about the myriad systems in which an ABC ATPase and a Toprim domain work in concert in defense systems. Future studies will surely uncover in more detail how OLD proteins have been leveraged by organisms to defend themselves.

## Author contributions

KA: Writing – original draft, Writing – review and editing. BT-S: Conceptualization, Visualization, Writing – original draft, Writing – review and editing.

## Funding

The author(s) declare financial support was received for the research, authorship, and/or publication of this article. This work was supported by Davidson College.

## Conflict of interest

The authors declare that the research was conducted in the absence of any commercial or financial relationships that could be construed as a potential conflict of interest.

## Publisher’s note

All claims expressed in this article are solely those of the authors and do not necessarily represent those of their affiliated organizations, or those of the publisher, the editors and the reviewers. Any product that may be evaluated in this article, or claim that may be made by its manufacturer, is not guaranteed or endorsed by the publisher.
